# Adapting a Green Activity Program With Older Black People Living With Memory Challenges

**DOI:** 10.1093/geroni/igaf122.1201

**Published:** 2025-12-31

**Authors:** Christopher Carey, Lola Sample, Laura Gitlin, Jaroslaw Harezlak, Richard Holden, NiCole Keith, Kathleen Unroe, Rebecca Lassell

**Affiliations:** Indiana University School of Medicine, Indianapolis, Indiana, United States; Indiana University School of Public Health Bloomington, Bloomington, Indiana, United States; Drexel University, Philadelphia, Pennsylvania, United States; Indiana University Bloomington, Bloomington, Indiana, United States; Indiana University Bloomington, Bloomington, Indiana, United States; Indiana University School of Public Health Bloomington, Bloomington, Indiana, United States; Indiana University School of Medicine, Indianapolis, Indiana, United States; Indiana University Bloomington, Bloomington, Indiana, United States

## Abstract

Few physical activity programs are designed with Black older adults living with mild cognitive impairment and dementia (memory challenges) to address their needs. Applying the Cultural Adaptation Process model and the Framework for Modification and Adaptations, we sought to adapt a nature or ‘Green’ Activity Program for Black people living with memory challenges (PLMC) and study partners. Participants were recruited from Eskenazi Health in Indianapolis, Indiana. A modified 5-step co-design process occurred with six 90-minute sessions with four teams separately (n = 21 total, n = 5-6 each). Participants were Black PLMC (n = 5), Black study partners (n = 6), outdoor professionals (n = 5), and healthcare providers (n = 5). Recorded design sessions were analyzed. Challenges for PLMC included: 1) noise (construction, traffic), 2) weather 3) aches and pain; Study partners: 1) health challenges/behaviors, 2) transportation/accessibility; 3) finances. Solutions identified by PLMC were 1) wearing headphones, 2) weather appropriate clothing, 3) activity modifications; Study partners: 1) accessibility/motivation, 2) transportation services and convenience, and 3) free/lower cost activities. Surface cultural adaptions (language) included preferred terms of ‘memory challenges’ for dementia and ‘Black.’ Deep cultural adaptations (values/beliefs) included adding family, awareness/exposure to nature activities. ‘Content’ adaptations included preferred nature activities of water Zumba, fishing, and camping. ‘Contextual’ modifications for program delivery were a ‘green activity pill box’ to schedule weekly activities as a visual reminder, a buddy system, and monetary incentives for motivation. Referral pathways included direct referrals in electronic health records, or in-person from healthcare providers. Findings can guide piloting the program and cultural adaptation of other interventions.

